# The prevalence rates of major chronic diseases in retired and in-service Chinese military officers (2000–2016): a meta-analysis

**DOI:** 10.1186/s40779-017-0148-z

**Published:** 2018-01-30

**Authors:** Thermite Mara, Long-Teng Ma, Shuo Wang, Ling Wang, Fan Yang, Jia-Hui Song, Yi-Chun Cao, Jian-Hua Yin, Guang-Wen Cao

**Affiliations:** Department of Epidemiology, Second Military Medical University, Shanghai, 200433 China

**Keywords:** Chronic diseases, Meta-analysis, Hypertension, Hyperlipidemia, Diabetes, Cerebrovascular disease, Chronic obstructive pulmonary disease

## Abstract

**Background:**

Chronic diseases cause a tremendous burden to the military medical system. However, the prevalence rates of major chronic diseases among military officers remain unclear in China.

**Methods:**

China National Knowledge Infrastructure (CNKI), Wanfang Database, VIP Database for Chinese Technical Periodicals (VIP), PubMed and Web of Science were searched for studies (from 2000 to 2016) concerning 6 major chronic diseases: hypertension, hyperlipidemia, diabetes mellitus, heart diseases, cerebrovascular diseases, and chronic obstructive pulmonary diseases (COPD) in Chinese military officers following strict inclusion and exclusion criteria. Three researchers independently extracted data from the included studies, and a fourth researcher reviewed and solved every disagreement. Statistical analysis was performed with STATA 14.0 and R 3.3.2. Heterogeneity was evaluated by the *I*^*2*^ value. A random effect model was performed to combine the heterogeneous data. The Egger test was performed to test the publication bias.

**Results:**

A total of 90,758 military officers derived from 75 articles were pooled together. Publication bias was only observed in 37 studies reporting heart disease (*P*_Egger test_ = 0.01). The overall prevalence rates of hypertension, hyperlipidemia, diabetes mellitus, heart diseases, cerebrovascular diseases, and COPD were 46.6% (95% CI 41.8–51.5%), 30.9% (26.4–35.7%), 20.7% (16.5–25.7%), 48.2% (41.7–54.9%), 20.2% (14.8–26.9%) and 16.6% (12.9–21.0%), respectively. The prevalence rates of hypertension, diabetes, heart disease, cerebrovascular disease, and COPD, rather than hyperlipidemia, increased with age in Chinese military officers. Heart diseases (*P*_*Q-test*_ < 0.001) and hypertension (*P*_*Q-test*_ < 0.001) increased sharply in retired officers compared with officers in service. Cerebrovascular disease was more frequent in Northern Theater Command than in any other theater command (*P*_*Q-test*_ < 0.001).

**Conclusions:**

Major chronic diseases heavily affect Chinese military officers, especially retirees. Medical intervention should be enforced on the prevention of cerebrovascular diseases in those working in cold areas in the north, as well as hypertension and heart diseases in retirees.

## Background

With the development of medical services in China, group 1 (communicable, maternal, perinatal, and nutritional conditions) and group 3 (injury) diseases have decreased remarkably, and group 2 diseases (non-communicable chronic diseases) have gradually increased (group 1, 2 and 3 are standard classification of diseases for ICD-10) [1]. Chronic diseases, which are characterized by high prevalence, long time course, and refraction to treatment, place a tremendous burden on the medical service systems of China.

The prevalence rates of major chronic diseases among civilians on the national level have been investigated and reported, while the prevalence rates of chronic diseases among whole military officers remain unclear [2–4]. Many previous small-scale epidemiological studies on the prevalence of chronic diseases in military officers have been performed since the 1960s. However, the results of these small-scale studies are poorly generalizable, because their target population is from different age groups or different theater commands. To obtain a representative estimate of the prevalence rates of chronic diseases among most Chinese military officers, we performed this meta-analysis.

In this study, we searched literature databases for studies concerning chronic disease prevalence rates in Chinese military officers. We focused on six major chronic diseases, including hypertension, hyperlipidemia, diabetes mellitus, heart diseases, cerebrovascular diseases, and chronic obstructive pulmonary disease (COPD). We conducted subgroup analyses between retired officers and those in service, among different age groups, and among the 5 theater commands.

## Methods

### Search strategy

Three authors searched the following databases independently: China National Knowledge Infrastructure (CNKI), Wanfang Database, VIP Database for Chinese Technical Periodicals (VIP), PubMed and Web of Science. The following search items and their synonyms were used: “Hypertension”, “Hyperlipidemia”, “Diabetes Mellitus”, “Heart Diseases”, “Cerebrovascular Diseases”, “Chronic Pulmonary Obstructive Diseases”, “China Military”, and “China Military Retirement”. Relevant literature was found in CNKI, Wanfang and VIP but not in PubMed and Web of Science.

### Inclusion and exclusion criteria

The inclusion criteria were as follows: 1) the study population included in-service or retired Chinese military officers; 2) the study design was longitudinal or cross-sectional; 3) the study population and the number of patients with each disease was clearly described; 4) the studies showed the age groups of the study population; 5) the diseases were diagnosed according to the national criteria (National criteria meant clinical guidelines published by the National Health and Family Planning Commission of the People’s Republic of China, which were consistent with criteria published by the World Health Organization.); 6) the studies were published from Jan 1, 2000 to Dec 31, 2016.

The exclusion criteria were as follows: 1) studies that had improper age divisions (the difference between the upper age and the lower age is more than 40 in any age group); 2) studies that surveyed hospitalized patients rather than the ambulatory population; 3) studies that were not carried out in mainland China; 4) reviews and editor reports.

### Data extraction

**Fig. 1:**
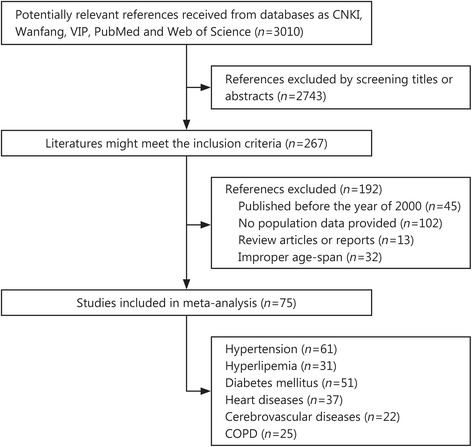
The procedure for selecting studies. COPD. Chronic obstructive pulmonary disease

According to the inclusion and exclusion criteria, Thermite MARA, Longteng Ma, and Shuo Wang read all the articles and extracted the following data independently: title, author, the number of target populations and patients, age groups, study area, and career status (in-service or retired). We mapped the included populations to the 5 theater commands (Central, Western, Eastern, Northern, and Southern Theater Commands). Fan Yang reviewed every disagreement. Few studies provide data about the complications of chronic diseases, so we did not analyze the prevalence rates of complications in chronic diseases. The study selection process and reasons for exclusions are depicted in Fig. 1.

### Statistical analysis

STATA 14.0 StataCorp LLC, College Station, TX, USA) and R 3.3.2 with the “meta” package were utilized for data analyses. For each disease, the prevalence rates from the different studies were combined and the 95% confidence interval (95% CI) was calculated. Then, homogeneity tests were carried out (*I*^*2*^ > 50% was chosen as the threshold of significant heterogeneity). When heterogeneity existed, a random effects model was selected to pool the prevalence data from the different studies. Subgroup analyses were performed to compare the prevalence rates of the chronic diseases among officers in different age groups (30–59, 60–79, >80 years old), and in different theater commands. A *Q*-test based on the analysis of variance was performed in the subgroup analyses to determine whether the difference between the subgroups was significant or not. In analyses that involved more than 10 studies, an Egger test was performed to test the publication bias, and *P* < 0.05 was considered statistically significant.

## Results

### Overall analysis

A total of 75 studies were eventually included in this study (Table 1 and Appendix Table 2). Of those, 61 articles reported hypertension, 31 reported hyperlipidemia, 51 reported diabetes mellitus, 37 reported heart diseases, 22 reported cerebrovascular diseases, and 25 reported COPD. A total of 90,758 officers were surveyed in the 75 studies, and all of them were older than 30 years old. Among these military officers, 28,794 were diagnosed with hypertension, 11,936 were diagnosed with hyperlipidemia, 11,752 were diagnosed with diabetes, 14,644 were diagnosed with heart diseases, 2064 were diagnosed with cerebrovascular diseases, and 3670 were diagnosed with COPD. As shown in Table 1, the overall prevalence rate of hypertension, hyperlipidemia, diabetes mellitus, heart diseases, cerebrovascular diseases, and COPD was 46.65% (95% CI 41.83–51.52%), 30.86% (26.41–35.70%), 20.7% (16.47–25.75%), 48.26% (41.70–54.89%), 20.16% (14.75–26.94%), and 16.60% (12.95–21.03%), respectively. As heterogeneity existed, the data were pooled using a random effect model.

**Table 1:** Overall analysis of six chronic diseases

Disease	Number of studies	Included References	In-service/Retired/Both	Prevalence Rate	*I* ^*2*^	P value of Egger Test
Hypertension	61	[12–72]	9/46/6	46.65% (41.83%-51.52%)	99.27%	0.11
Hyperlipidemia	31	[14, 16, 18, 19, 21, 26, 27, 30–32, 34, 36–39, 42–44, 46–49, 58, 62–65, 67, 70–73]	4/23/4	30.86% (26.41%-35.7%)	98.89%	0.67
Diabetes mellitus	51	[14, 15, 18, 19, 20, 21, 22, 24–31, 33, 34, 36–46, 48–51, 53, 54, 57, 59, 63–68, 70–72, 74–79]	4/43/4	20.73% (16.47%-25.75%)	99.39%	0.88
Heart diseases	37	[14, 18–22, 25–29, 33, 34, 36, 38, 39, 40–46, 48, 49, 50–54, 59, 62, 64, 67, 70, 71, 72]	2/30/5	48.26% (41.7%-54.89%)	99.25%	0.01
Cerebrovascular disease	22	[15, 20, 21, 27–29, 33, 34, 43, 48, 50, 52, 54, 59, 71, 80–86]	1/21/0	20.16% (14.75%-26.94%)	99.02%	0.93
COPD	25	[16, 18, 21, 25, 27, 28, 29, 30, 33, 34, 39, 40, 41, 44–46, 48, 50, 51, 53, 54, 66, 67, 70,71]	0/23/2	16.60% (12.95%-21.03)	98.28%	0.39

### The prevalence rates of chronic diseases among the subgroups

#### The prevalence rates of chronic diseases among the different age groups

**Fig. 2:**
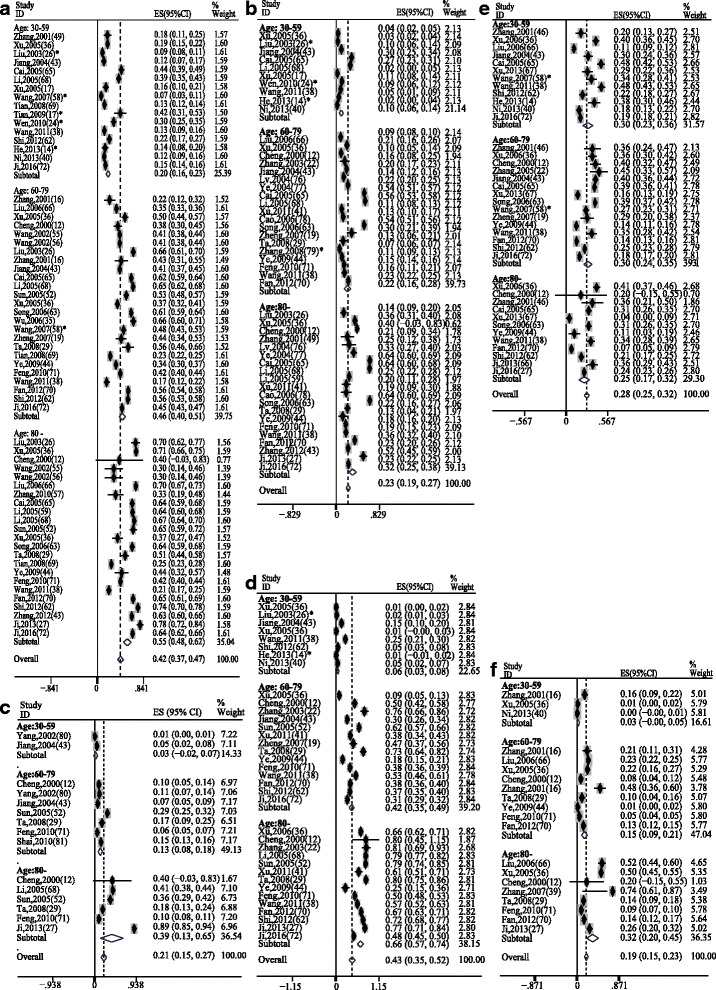
Meta-analysis on the prevalence of the major chronic diseases by age group. **a**. Hypertension; **b**. Diabetes mellitus; **c**. Cerebrovascular diseases; **d**. Heart diseases; **e**. Hyperlipidemia; **f**. Chronic obstructive pulmonary disease. * study was carried out in active military officers

**Fig. 3:**
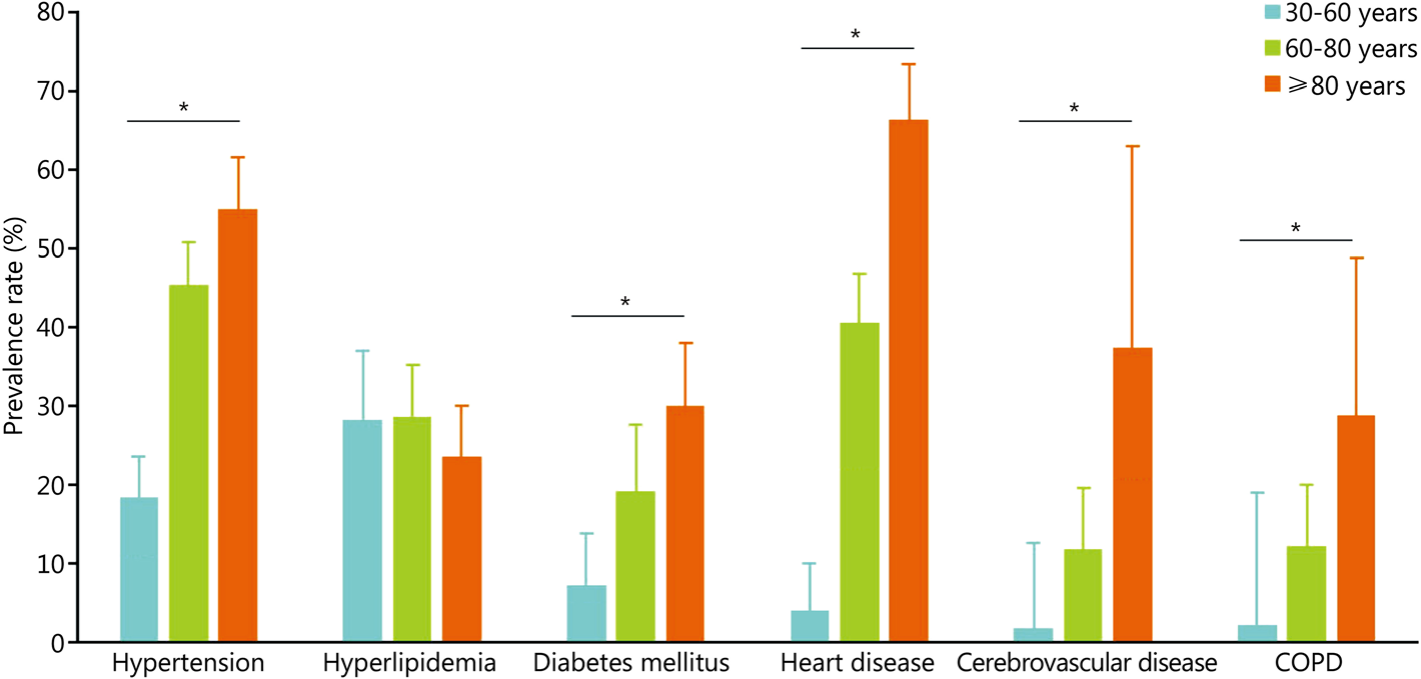
The prevalence rates of the major chronic diseases of military officers in different age groups. Bars indicate the 95% confidence intervals (CI) in this plot. * *P* < 0.05. COPD. Chronic obstructive pulmonary disease

To increase the comparability, we set 3 age groups with sufficient participants in each group: 30–59 years (13,415 participants), 60–79 years (32,873 participants) and older than 80 years (11,998 participants). Meta-analyses on age-subgroups were conducted to reduce the effect of age structure on the prevalence. A general increasing trend for the prevalence was shown in the forest plots for most of the diseases (Fig. 2). The prevalence rates of hypertension (*P* < 0.001), diabetes (*P* < 0.001), heart diseases (*P* < 0.001), cerebrovascular diseases (*P* = 0.005), and COPD (*P* = 0.028) differed significantly between age groups. However, this phenomenon was not found in the prevalence rate of hyperlipidemia (*P* = 0.457, Fig. 3). Thus, the prevalence rates of chronic diseases increased rapidly with age except for hyperlipidemia.

#### The prevalence rates of chronic diseases between retired officers and in-service officers

**Fig. 4:**
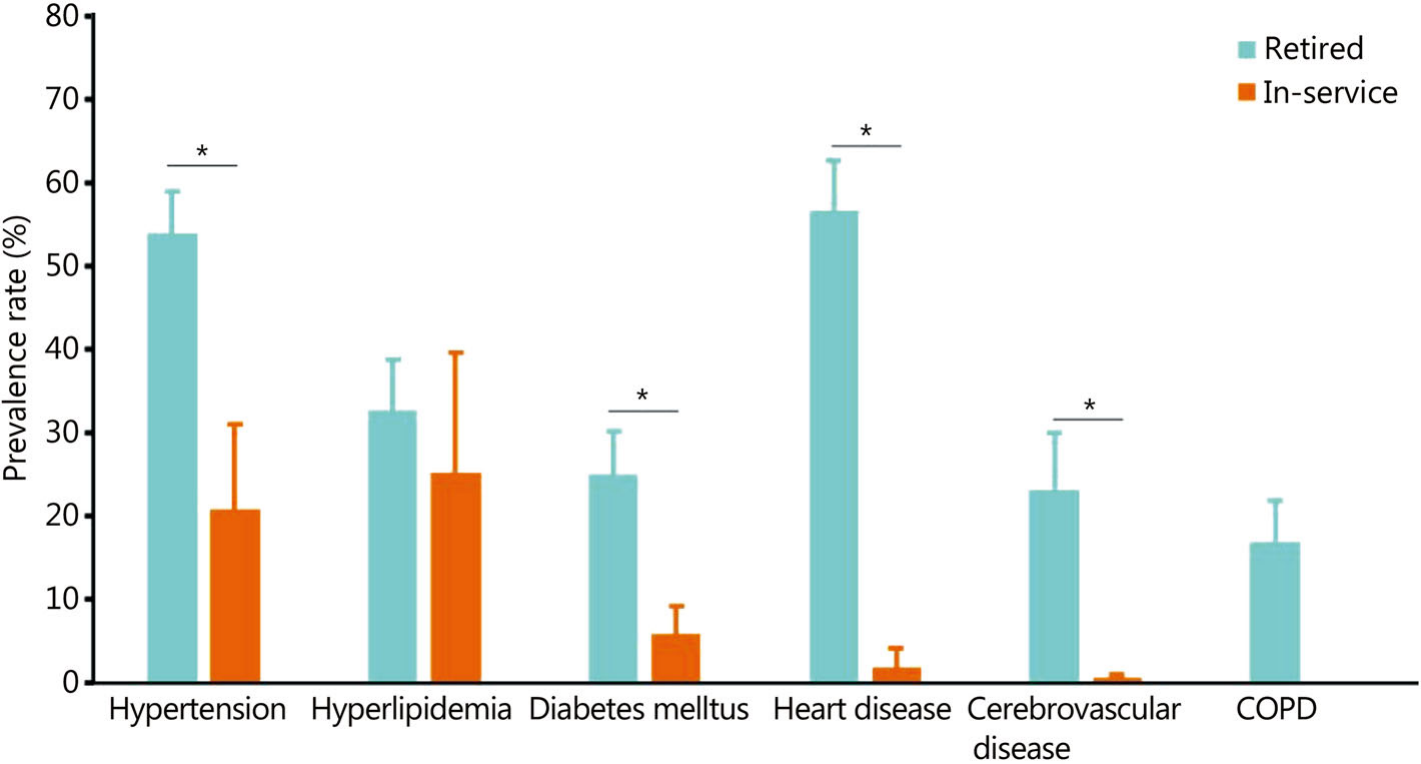
The prevalence rates of the major chronic diseases of retired military officers and active military officers. Bars indicate the 95% confidence interval (CI) in this plot. * *P* < 0.05. COPD. Chronic obstructive pulmonary disease

Among the 75 articles included, 62 reported data from retired officers and 19 reported data from in-service officers. The prevalence rates of heart diseases, hypertension, cerebrovascular diseases, and diabetes mellitus in retired officers were significantly higher than those in in-service officers. The prevalence rate of heart disease in retired officers was 54.7 percentage points higher than in-service officers (in-service officers vs. retired officers: 1.9% *vs*. 56.6%). The numbers were 32.9 for hypertension (in-service officers vs. retired officers: 20.9% *vs*. 53.8%), 22.5 for cerebrovascular diseases (in-service officers vs. retired officers: 0.6% *vs*. 23.1%), and 19.7 for diabetes (in-service officers vs. retired officers: 5.3% *vs*. 25.0%). No significant differences in the prevalence of hyperlipidemia were found between retired officers and those in service (in-service officers vs. retired officers = 25.1% *vs*. 32.6%, Fig. 4). We did not find any article reporting the prevalence rate of COPD among in-service officers. The numbers of studies concerning hypertension, hyperlipidemia, diabetes mellitus, heart diseases, cerebrovascular disease, and COPD for in-service military officers were 9, 4, 4, 2, 1, and 0, respectively. The number of studies among active officers was not sufficient to perform an Egger test.

#### The prevalence rates of chronic diseases among the 5 theater commands

**Fig. 5:**
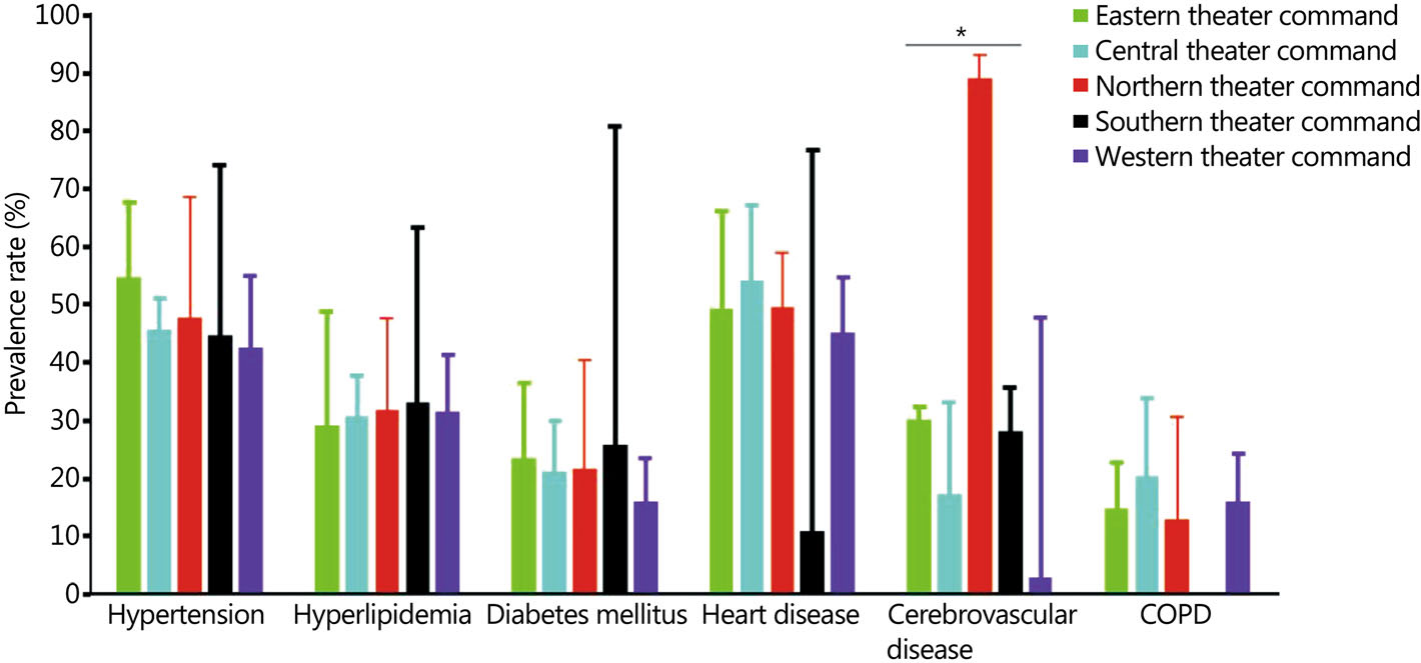
The prevalence rates of the major chronic diseases of military officers in 5 different theater commands. Bar indicates 95% confidence interval (CI) in this plot. * *P* < 0.05. COPD. Chronic obstructive pulmonary disease

We analyzed the cities where the articles were carried out and matched these cities to the 5 theater commands. A total of 12 articles were from the Eastern Theater Command, 30 articles from Central Theater Command, 7 articles from Northern Theater Command, 8 articles from Southern Theater Command, and 18 articles from Western Theater Command. Significant differences between the theater commands were observed for the prevalence rate of cerebrovascular disease (*P* < 0.001, Fig. 5).

### Publication bias

Publication bias was a type of bias that occurred when the research outcome influenced the decision whether to publish the study. We made funnel plots and performed Egger tests to explore the publication bias. No significant result was observed except for heart disease. Inspection of the funnel plot showed significant bias with a positive skew for the prevalence rate of heart disease, which indicated that the result of heart diseases should be carefully interpreted (Table 1).

## Discussion

Our study summarized the prevalence rates of six chronic diseases among Chinese military officers older than 30 years old. We found that the prevalence rates of chronic disease increased with age, except for the prevalence rate of hyperlipidemia. We also found that cerebrovascular disease was more frequent in Northern Theater Command.

Figure 3 shows that the prevalence rates of chronic diseases differ significantly among age groups. Increasing trends were pronounced in COPD, heart diseases, diabetes, and cerebrovascular diseases. Interestingly, the prevalence of hyperlipidemia did not differ significantly among the different age groups, possibly because hyperlipidemia may be caused by food structure or physical activity or both [5]. Previous studies have demonstrated that hyperlipidemia is correlated with hypertension, heart disease, and diabetes mellitus [4, 6].

The prevalence rates of chronic disease were higher in retired military officers than those in active military officers. Age structure might be the major cause of this difference. Here, we demonstrated that evident increases were observed in heart diseases and hypertension in retired officers. Lifestyle changes after retirement might contribute to this increase. Thus, more attention should be paid to heart disease and hypertension prevention after retirement.

The prevalence of chronic disease, except cerebrovascular diseases, was not different among the 5 Theater Commands. This suggested that the major risk factors for these chronic diseases were not different in the 5 theater commands. However, the prevalence rate of cerebrovascular diseases was much higher in the Northern Theater Command, which might be illustrated by the high-salt diet and cold climate in Northern China. High salt intake has been shown to be associated with blood pressure and stroke [7]. The highly variable climate in Northern China might also contribute to the higher prevalence of cerebrovascular diseases. Large population-based studies showed that the changeable climate was a risk factor for cerebrovascular diseases [8]. Although the risk factors for the chronic diseases were not different in the 5 theater commands, the risk factors promoting the development of cerebrovascular diseases, such as high salt intake and cold climate, are specific in the Northern Theater Command.

The prevalence rates of hypertension, diabetes mellitus, and COPD in this study are close to the corresponding data at the national level [2–4]. Of note, the estimated hypertension prevalence in the natural population in China was 47% in the 65–74 age group, while it was 46% in military officers in the 60–80 age group [9]. In terms of diabetes, the estimated prevalence was 23% in the 60–69 age group, while the number was 22% in military officers [4]. The same was true for COPD (civilian vs. military officers = 12% vs. 15%, in 60–69 age group) [10]. However, the prevalence rate of hyperlipidemia in our study is lower than that in a nationwide meta-analysis (civilian vs. military officers = 41.9% vs. 30.9%) [11]. As shown in our study, the prevalence of hyperlipidemia did not increase with age. For military officers, physical training in their youth might reduce the risk of hyperlipidemia. To date, no cerebrovascular prevalence rate has been reported nationwide.

There are many centers for disease control and prevention and military hospitals in the Chinese military system, which provides free medical service to both active and retired military officers. However, it seems that these authorities do not significantly reduce the prevalence rates of chronic disease in Chinese military officers. Our study indicates that the primary prevention of major chronic diseases in Chinese military system should be improved. Intervention on the unhealthy lifestyle of military officers is necessary for the early prevention of chronic diseases. In addition, it is necessary to promote a low-salt diet in Northern China. These recommendations are suitable for both active military officers and retired military officers and should be realized by the corresponding healthcare providers.

### Limitation and strength

Our study has several limitations. First, there was heterogeneity between the different studies. Second, as the age structure of the study population was different from that of active military officers, the overall prevalence rates must be interpreted with caution. Third, publication bias existed in the heart disease studies. Although our study has advantages, to the best of our knowledge, this is the first study investigating the prevalence rates and age-specific prevalence rates of major chronic diseases among Chinese military officers on a whole-military scale. We also conducted sub-group analyses to evaluate the prevalence rates among age-groups, career status, and theater commands. An overview of disease burden among the military officers was important for optimizing military medical service.

## Conclusion

Our study provides a comprehensive overview and provides the prevalence rates of chronic diseases in Chinese military officers older than 30 years old. The prevalence rates of chronic disease among Chinese military officers were consistent with those among civilians, except for hyperlipidemia. The prevalence of hypertension, diabetes, heart diseases, cerebrovascular diseases, and COPD increased with increasing age, whereas this increasing trend was not shown in hyperlipidemia. The prevalence of cerebrovascular diseases was extremely high in the Northern Theater Command, which might be attributed to diet and highly variable climate. Overall, this research might help optimize the prevention and control of chronic diseases in military systems worldwide.

## Appendix

**Table 2:** Characteristics of included studies

Status	Theater command	Author	Year	Age groups	Population	Hypertension	Hyperlipidemia	Diabetes mellitus	Heart diseases	Cerebrovascular diseases	COPD
in-service	central	Cheng et al. [12]	2000	-	54	10	-	-	-	-	-
in-service	central	Wu et al. [13]	2011	-	124	11	-	-	-	-	-
in-service	central	He et al. [14]	2013	1	137	20	54	7	1	-	-
retired	southern	Pan et al. [15]	2013	-	68	23	-	35	-	32	-
retired	western	Zhang et al. [16]	2001	1,2	185	37	29	-	-	-	33
in-service	western	Tian et al. [17]	2009	1	95	41	-	-	-	-	-
retired	central	Zan et al. [18]	2003	2,3	106	43	45	20	84	-	62
retired	eastern	Zheng et al. [19]	2007	2,	98	44	29	30	46	-	-
retired	central	Zhao et al. [20]	2011	-	89	46	-	24	55	30	-
retired	central	Chen et al. [21]	2000	2,3	168	65	68	18	87	13	14
retired	central	Zhang et al. [22]	2005	-	99	69	-	36	63	-	-
in-service	western	Xie et al. [23]	2016	-	280	81	-	-	-	-	-
in-service	central	Wen et al. [24]	2010	1	319	98	-	36	-	-	-
retired	western	Lv et al. [25]	2008	-	149	105	-	50	100	-	31
in-service	southern	Liu et al. [26]	2003	1	1304	126	142	42	30	-	-
retired	northern	Ji et al. [27]	2013	3	172	138	64	92	135	111	45
retired	central	Wang et al. [28]	2015	-	175	151	-	90	128	56	47
retired	central	Ta et al. [29]	2008	2,3	288	155	-	57	228	37	36
retired	eastern	Jing et al. [30]	2010	-	227	172	176	187	-	-	91
retired	central	Zhang et al. [31]	2016	-	477	178	292	65	-	-	-
in-service	western	Ma et al. [32]	2008	-	1053	184	353	-	-	-	-
retired	central	Fang et al. [33]	2013	-	268	186	-	46	212	63	78
retired	central	Yang et al. [34]	2013	-	267	190	107	95	199	113	62
retired	southern	Wu et al. [35]	2006	2,	282	194	-	-	-	-	-
retired	central	Xu et al. [36]	2005	1,2,3	690	226	124	70	229	-	-
retired	northern	Zhang et al. [37]	2013	-	356	229	194	161	-	-	-
in-service/retired	northern	Wang et al. [38]	2011	1,2,3	1516	234	685	178	569	-	-
retired	northern	Zhang et al. [39]	2007	-	1094	236	138	125	412	-	50
in-service/retired	eastern	Ni et al. [40]	2013	1	811	240	-	48	127	-	46
retired	western	Xu et al. [41]	2011	-	534	356	-	58	317	-	88
retired	southern	Huang et al. [42]	2016	-	433	268	256	250	174	-	-
retired	central	Jiang et al. [43]	2004	1,2	777	270	300	139	207	37	-
retired	eastern	Ye et al. [44]	2009	2,3	760	271	107	87	143	-	9
retired	eastern	Wang et al. [45]	2016	-	328	282	-	97	265	-	68
retired	central	Li et al. [46]	2008	-	418	282	157	122	275	-	104
retired	eastern	Wu et al. [47]	2003	-	1251	313	709	-	-	-	-
retired	western	Fei et al. [48]	2002	-	2086	332	804	152	581	8	141
retired	western	Song et al. [49]	2005	-	764	337	301	166	275	-	-
retired	eastern	Chen et al. [50]	2013	-	478	348	-	108	239	101	70
retired	northern	Xu et al. [51]	2011	-	534	356	-	58	317	-	88
retired	eastern	Sun et al. [52]	2005	2,3	716	416	-	-	485	159	-
retired	western	Song et al. [53]	2005	-	534	423	-	58	317	-	88
retired	eastern	Sun X et al. [54]	2011	-	745	427	-	118	494	161	235
retired	central	Wang et al. [55]	2002	2,3	1174	490	-	-	-	-	-
retired	central	Wang et al. [56]	2002	2,3	880	307	-	-	-	-	-
retired	western	Zhang et al. [57]	2010	3	933	603	-	219	-	-	-
in-service	central	Wang et al. [58]	2007	1,2	1598	635	250	-	-	-	-
retired	central	Li et al. [59]	2005	3	974	673	-	248	784	288	-
retired	western	Zhou et al. [60]	2008	-	1399	740	-	-	-	-	-
retired	southern	Xie et al. [61]	2011	2,3	1240	878	-	-	-	-	-
in-service/retired	northern	Shi et al. [62]	2012	1,2,3	1891	1060	464	-	766	-	-
retired	central	Song et al. [63]	2006	2,3	1687	1077	643	975	-	-	-
retired	eastern	Cha et al. [64]	2012	-	1244	1080	208	396	769	-	-
retired	central	Cai et al. [65]	2005	1,2,3	2021	1225	900	1072	-	-	-
retired	eastern	Liu et al. [66]	2006	2,3	2862	1250	-	309	-	-	828
in-service/retired	western	Xu et al. [67]	2013	1,2,3	2457	1284	1151	531	759	-	718
retired	central	Li et al. [68]	2005	1,2,3	2172	1325	-	1141	-	-	-
in-service/retired	western	Tian et al. [69]	2008	1,2,3	6002	1397	-	-	-	-	-
retired	western	Fan et al. [70]	2012	2,3	2668	1595	348	710	1201	-	364
retired	central	Feng et al. [71]	2010	2,3	3889	1890	-	730	1887	231	274
in-service/retired	central	Ji et al. [72]	2016	1,2,3	7788	3072	1925	490	1684	-	-
retired	southern	Ze et al. [73]	2003	-	2047	-	913	-	-	-	-
retired	eastern	Zheng et al. [74]	2012	-	705	-	-	290	-	-	-
retired	central	Fan et al. [75]	2003	-	1776	-	-	323	-	-	-
retired	central	Lv et al. [76]	2004	2,3	1217	-	-	183	-	-	-
retired	western	Ye et al. [77]	2004	2,3	1630	-	-	398	-	-	-
retired	western	Cao et al. [78]	2006	2,3	395	-	-	58	-	-	-
in-service	western	Zhang et al. [79]	2008	2	10207	-	-	754	-	-	-
retired	central	Yang et al. [80]	2002	1,2	325	-	-	-	-	28	-
in-service	central	Shai et al. [81]	2002	-	3105	-	-	-	-	16	-
retired	central	Zhou et al. [82]	2008	-	1245	-	-	-	-	43	-
retired	southern	Duan et al. [83]	2012	-	1024	-	-	-	-	150	-
retired	southern	He et al. [84]	2013	-	1498	-	-	-	-	220	-
retired	western	Sai et al. [85]	2010	2	1232	-	-	-	-	129	-
retired	central	Wu et al. [86]	2014	-	164	-	-	-	-	38	-

Age groups : 1="30-59 years group", 2="60-79 years group", 3="80- years group". "-": data not available

## Abbreviations

COPD: Chronic obstructive pulmonary disease
CNKI: China National Knowledge Infrastructure
VIP: VIP Database for Chinese Technical Periodicals
